# Genome-Wide Association Study Reveals Constant and Specific Loci for Hematological Traits at Three Time Stages in a White Duroc × Erhualian F_2_ Resource Population

**DOI:** 10.1371/journal.pone.0063665

**Published:** 2013-05-17

**Authors:** Zhiyan Zhang, Yuan Hong, Jun Gao, Shijun Xiao, Junwu Ma, Wanchang Zhang, Jun Ren, Lusheng Huang

**Affiliations:** Key Laboratory for Animal Biotechnology of Jiangxi Province and the Ministry of Agriculture of China, Jiangxi Agricultural University, Nanchang, China; Wageningen UR Livestock Research, Netherlands

## Abstract

Hematological traits are important indicators of immune function and have been commonly examined as biomarkers of disease and disease severity in humans. Pig is an ideal biomedical model for human diseases due to its high degree of similarity with human physiological characteristics. Here, we conducted genome-wide association studies (GWAS) for 18 hematological traits at three growth stages (days 18, 46 and 240) in a White Duroc × Erhualian F_2_ intercross. In total, we identified 38 genome-wide significant regions containing 185 genome-wide significant SNPs by single-marker GWAS or LONG-GWAS. The significant regions are distributed on pig chromosomes (SSC) 1, 4, 5, 7, 8, 10, 11, 12, 13, 17 and 18, and most of significant SNPs reside on SSC7 and SSC8. Of the 38 significant regions, 7 show constant effects on hematological traits across the whole life stages, and 6 regions have time-specific effects on the measured traits at early or late stages. The most prominent locus is the genomic region between 32.36 and 84.49 Mb on SSC8 that is associated with multiple erythroid traits. The *KIT* gene in this region appears to be a promising candidate gene. The findings improve our understanding of the genetic architecture of hematological traits in pigs. Further investigations are warranted to characterize the responsible gene(s) and causal variant(s) especially for the major loci on SSC7 and SSC8.

## Introduction

In the immune system, hematological traits include three components: leukocytes (white blood cells, WBCs), erythrocytes (red blood cells, RBCs) and platelets. All of these components represent important parameters of immune capacity of individuals [1]. Hematological related cells in the peripheral blood execute a range of functions including the transport of oxygen, innate and adaptive immunity, vessel wall surveillance, homeostasis and wound repair. As blood incessantly flows within the circulatory system around organs and tissues, it can reflect any slightly abnormal changes in the body rapidly by testing the changes of cells number and (or) cells volume. Deviations outside normal ranges for these parameters are indicative of different kinds of disorders including cancer and cardiovascular, metabolic, infectious and immune diseases [2]. Measurements of erythrocytes within the blood are becoming a routine examination to uncover various hematological related disorders.

The count and volume of cellular elements in circulating blood are highly heritable and vary considerably among individuals [3]–[5]. In human, genome-wide association studies (GWAS) have identified >60 loci associated with hematological parameters in individuals of European ancestry, Japanese population, and African Americans [2], [6]–[12]. However, these polymorphisms explain only a small fraction of the genetic variance in hematological traits. This is so called “missing heritability” [13]. Well-designed study in animal model is an efficient way to identify additional genetic factors contributing to complex phenotypic variance. The domestic pig is a large-animal model for human genetic diseases due to its high degree of similarity with human physiological characteristics. Identification of responsible genes and causal variants for hematological traits in pigs would benefit researches on human medicine.

So far, 239 quantitative trait loci (QTL) for swine hematological traits have been reported by linkage mapping in the AnimalQTLdb database [14], but the confidence intervals of these QTL are generally large (>20 cM) and harbor hundreds of functional genes, thereby hampering the characterization of plausible candidate genes. Compared to traditional QTL mapping strategies, GWAS based on high-density markers is a more powerful tool to identify genomic regions for phenotypic traits. To our knowledge, only two very recent studies have reported the GWAS for hematological parameters in pigs [15], [16]. The two studies identified 10 and 62 genome-wide significant loci for hematological traits. However, only one locus for RDW on pig chromosome (SSC) 12 was consistently detected in the two studies, implying the complexity and heterogeneity of hematological traits.

In our previous studies, we conducted a whole genome linkage mapping in a White Duroc × Erhualian F_2_ resource population using 183 microsatellite markers, and identified a number of QTL affecting hematological traits measured at 3 growth stages [17], [18]. To fine map the identified QTL and uncover new genetic variants associated with hematological traits, we herein performed GWAS on the F_2_ resource population using the PorcineSNP60 Genotyping BeadChip technology (Illumina, USA). The experimental data are available upon the readers’ request.

## Materials and s

### Ethics Statement

All the procedures involving animals are in compliance with the care and use guidelines of experimental animals established by the Ministry of Agriculture of China. The ethics committee of Jiangxi Agricultural University specifically approved this study.

### Animals and Phenotypic Measurements

A detail description of the White Duroc × Erhualian F_2_ resource population and phenotype recording have been presented in our previous publications [17]–[19]. Briefly, the three-generation resource population comprising 1912 F_2_ individuals was developed by crossing 2 White Duroc boars and 17 Erhualian sows. All animals were kept under a consistent standard pigpen and were fed with same diet at the experimental farm of Jiangxi Agricultural University. Eighteen hematological parameters were measured for 1449 individuals at three age stages: days 18, 46 and 240. Blood samples of 5 ml were collected from each animal and were directly injected into eppendorf tubes containing 30 ul of 20% EDTA in polybutadiene-styrene. A standard set of hematological data were recorded using a CD1700 whole blood analyzer (Abbott, USA) at the First Affiliated Hospital of NanChang University, China. The 18 hematological parameters include 7 baseline erythroid traits (hematocrit (HCT), hemoglobin (HGB), mean corpuscular hemoglobin (MCH), mean corpuscular hemoglobina concentration (MCHC), mean corpuscular volume (MCV), red blood cell count (RBC), and red blood cell volume distribution width (RDW) ), 7 leukocyte traits (granulocyte count (GRAN), granulocyte count percentage (GRAR), monocyte count (MON), monocyte count percentage (MONR), lymphocyte count (LYM), lymphocyte count percentage (LYMA), and white blood cell count (WBC) ), and 4 platelet traits (plateletcrit (PCT), platelet distribution width (PDW), platelet count (PLT), and mean platelet volume (MPV) ).

### Genotyping and Quality Control

Genomic DNA was extracted from ear tissues using a standard phenol/chloroform . All DNA samples were qualified and standardized into a final concentration of 20 ng/ul. A total of 1020 individuals in the F_2_ pedigree were genotyped for the Porcine SNP60 Beadchips on an iScan System (Illumina, USA) following the manufacturer’s protocol. Quality control was executed to exclude SNPs with call rate <95%, minor allele frequency (MAF) <5%, severely Hardy Weinberg disequilibrium (*P*<10E-5) and Mendelian inconsistency rate >10%. Moreover, individuals with missing genotypes >10% or Mendellian errors >5% were discarded from the data set.

### Statistical Analyses

#### Single-marker GWAS

The allelic difference of each SNP in phenotypic traits was tested using a general linear mixed model [20]–[22]. The model included a random polygenic effect, and the variance-covariance matrix was proportionate to genome-wide identity-by-state [23]. The formula of the model in mathematic is: 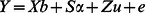, where Y is the vector of phenotypes, b is the estimator of fixed effects including sex and batch, α is the SNP substitution effect and u is the random additive genetic effect following multinomial distribution u ∼ N(0, **G**σ_α_^2^), in here G is the genomic relationship matrix that was constructed based on SNP markers as described in Eding et al [24], and σ_α_^2^ is the polygenetic additive variance. X, Z are the incidence matrices for b and u, S is incidence vector for α, e is a vector of residual errors with a distribution of N (0, **I**σ_e_^2^). All single-marker GWAS were conducted by GenABEL packages [25], [26]. The genome-wide significant threshold was determined by Bonferroni correction. It was defined as 0.05/N, where N is number of tested SNPs. In this study, the number of qualified SNPs is 39622 and the corresponding genome-wide threshold is 1.26e-6.

#### GWAS of time serials data

As all experimental individuals were recorded for hematological traits at three time stages (days 18, 46 and 240). We assumed that measurements at different stages in the same individual would be more correlated than those obtained from different individuals. To conceptualize this assumption, phenotypic records on the three age stages were analyzed together using a mixed effect approach to distinct the correlations within and/or among individuals [27], [28]. The model was similar to the above-mentioned single-marker GWAS model except that the phenotypic variance was partitioned to five parts rather than four parts: variance explained by SNPs, by fixed factors such as sex and batch, by polygenic effects, and by the time stage and by residual errors. The longitudinal GWAS were performed by LONG-GWAS [29] to adjust the variance and covariance structure among the three age stages. The genome-wide significant threshold was determined by Bonferroni correction as mentioned above.

#### Haplotype-based association studies and linkage disequilibrium (LD) analysis

A haplotype-based association study [30] was also performed to identify genomic regions associated with the tested hematological traits. Haplotypes corresponding to a predetermined number (K = 20) of hidden haplotype states [31] was conducted with a hidden Markov model via PHASEBOOK [32]. Association between phenotypes and the hidden haplotypes was detected under a generalized linear mixed framework that corrected population stratification by fitting a random polygenic effect. The mathematic formula of the mixed model was the same as the single-marker analysis, except that S was incidence matrices of hidden haplotype states rather than SNP genotypes and that the estimated haplotype effects were set as random effects. LD extents were estimated for significant SNPs using HAPLOVIEW [33].

## Results

### Phenotype Statistics and SNP Characteristics

Descriptive statistics of the measured traits in the current experimental population are presented in **Table 1**. Of the 7 baseline erythroid traits, 4 parameters including HCT, HGB, MCHC and RBC increased with age and one measurement (RDW) declined with age, while MCV and MCH first decreased and subsequently increased. Of the 7 leukocyte traits, GRAN and GRAR increased while LYMA decreased with age. The tendency of the other leukocyte traits varied irregularly. No consistent variation pattern was observed for platelet traits.

**Table 1:** Descriptive statistics of 18 hematological traits at three growth stages in the F_2_ resource population.

Trait	Abbreviation	Value (No.)		
		Day 18	Day 46	Day 240
Hematocrit (%)	HCT	0.30±0.07 (1447)	0.30±0.07 (1010)	0.41±0.05 (1010)
Hemoglobin (g/l)	HGB	95.55±22.83 (1444)	100.67±18.71 (1025)	137.35±16.08 (1025)
Mean corpuscular hemoglobin (pg)	MCH	19.34±3.21 (1445)	18.24±4.34 (1009)	19.28±1.49 (1009)
Mean corpuscular hemoglobin content (g/l)	MCHC	319.79±30.86 (1442)	341.41±51.29 (1009)	337.13±15.91 (1009)
Mean corpuscular volume (fl)	MCV	60.66±9.64 (1446)	52.70±8.13 (1019)	57.25±4.29 (1019)
Red blood cell count (10^12^)	RBC	4.92±0.94 (1447)	5.67±1.12 (1010)	7.14±0.87 (1010)
Red cell distribution width (%)	RDW	25.45±4.65 (1405)	24.10±6.34 (1002)	18.94±3.39 (1002)
Granulocyte count (10^9^)	GRAN	1.31±1.35 (1433)	2.96±2.91 (797)	7.47±4.31 (797)
Granulocyte count percentage(%)	GRAR	11.89±10.21 (1433)	15.77±13.28 (910)	41.71±20.69 (910)
Lymphocyte count (10^9^)	LYM	9.28±5.43 (1433)	13.76±5.4 (1024)	7.85±3.74 (1024)
Lymphocyte count percentage(%)	LYMA	79.29±13.79 (1448)	75.06±16.39 (1025)	46.29±17.85 (1025)
Monocyte count (10^9^)	MON	0.21±0.24 (550)	0.40±0.32 (754)	0.22±0.23 (754)
Monocyte count percentage(%)	MONR	1.96±2.11 (551)	2.19±1.68 (754)	1.30±1.35 (754)
White blood cell count (10^9^)	WBC	11.54±6.03 (1449)	18.45±5.97 (1024)	17.02±4.39 (1024)
Mean platelet volume (fl)	MPV	7.95±1.67 (666)	8.30±1.94 (760)	7.93±1.25 (760)
Plateletcrit (%)	PCT	0.44±0.23 (549)	0.75±1.08 (753)	0.23±0.1 (753)
Platelet distribution width (%)	PDW	15.53±2.44 (664)	14.43±1.85 (761)	15.49±0.94 (761)
Platelet count (10^9^)	PLT	559.26±219.65 (1386)	590.21±271.91 (1009)	295.67±117.75 (1009)

Values are shown in mean ± standard deviation; the numbers of recorded individuals are given in parentheses. Description of 7 erythroid traits in ∼1420 animals genotyped for 183 microsatellite markers has been shown in Zou (2008) *et al.* [18].

After quality control, 3077 SNPs with call rate <90%, 15711 SNPs with MAF <0.05, 6502 SNPs showing Hardy Weinberg disequilibrium (*P*<10E-5) and 208 markers exhibiting Mendelian inconsistency were excluded for further analyses. All individuals are qualified samples. A final set of 39622 SNPs on 1020 individuals were explored for the subsequent GWAS.

### Summary of Significant Loci Identified by GWAS

For GWAS on F_2_ populations, a cluster of significant SNPs would be typically detected at a significant locus due to large LD extent in such populations. If the distance between two genome-wide significant SNPs was small than 10 Mb, the two SNPs were treated as the same locus otherwise they were considered two independent loci in this study. According to this criterion, 13 genomic regions on 11 autosomes were strongly associated with blood cell parameters by single-marker GWAS or LONG-GWAS (**Table 2** **& Table S1**). The 13 regions harbor 185 significant SNPs and 91 annotated genes (data not shown). Of the 185 SNPs, 119 SNPs corresponding to 63 genes were evidenced by both association strategies. In the single-marker GWAS, 147 significant SNPs representing 78 genes were identified in comparison with 158 associated SNPs from 76 genes revealed by LONG-GWAS.

**Table 2:** Genome-wide significant loci associated with hematological traits by the single-marker analysis.

Trait 1	Top SNP	Chr 2	Pos (bp) 3	*P*-Value	Num 4	Interval (Mb) 5	Nearest gene 6
HCT240	ss107806758	7	35177641	8.98E-10	3	33.18–35.18	*SPDEF*
HCT240	ss131349087	7	45420438	1.69E-08	1	45.42	*CDC5L*
HCT240	ss131354973	7	56230077	5.90E-08	2	54.81–56.23	*EFTUD1*
HGB240	ss107806758	7	35177641	8.97E-11	8	33.18–35.25	*SPDEF*
HGB240	ss131349087	7	45420438	1.36E-07	1	45.42	*CDC5L*
HGB240	ss131354973	7	56230077	2.91E-07	1	56.23	*EFTUD1*
MCH18	ss131369009	8	44927836	5.03E-07	5	39.15–50.1	*TLL1*
MCH18	ss131083163	8	76480145	1.43E-07	2	76.48–77.78	*SHROOM3*
MCH240	ss131369293	8	50096834	1.17E-19	122	31.53–79.81	*PPID*
MCHC46	ss131260759	4	42489363	8.57E-07	1	42.49–42.49	*PGCP*
MCV18	ss131369293	8	50096834	2.66E-10	6	36.97–50.1	*PPID*
MCV18	ss131083163	8	76480145	2.77E-09	13	66.03–79.51	*SHROOM3*
MCV46	ss478938668	8	42150857	6.81E-07	1	42.15	*KIT*
MCV46	ss131076611	8	76669199	1.26E-06	1	76.67	*SHROOM3*
MCV240	ss131369293	8	50096834	1.08E-17	103	34.39–79	*PPID*
RBC18	ss131094241	8	49881116	1.40E-09	9	32.36–49.88	*RXFP1*
RBC18	ss107827400	8	66027033	3.12E-08	7	66.03–84.49	*TECRL*
RBC240	ss131369293	8	50096834	7.42E-11	14	34.39–50.1	*PPID*
RBC240	ss107906810	8	72567179	4.31E-08	5	66.03–74.51	*U6*
GRAR18	ss131368550	8	40777538	3.58E-07	1	40.78	*OCIAD1*
GRAR46	ss131364780	8	26310331	7.61E-07	1	26.31–26.31	*RPS18*
LYM18	ss131544979	17	2971217	2.64E-07	1	2.97	*ssc-mir-383*
WBC240	ss131344940	7	37288793	1.93E-07	2	37.23–37.29	*RAB44*
PDW46	ss107854351	13	14848132	3.94E-07	1	14.85	*LRRC3B*
PLT46	ss131296370	5	95971272	1.14E-08	1	95.97	DCN

1 Abbreviations of hematological traits are given in Table 1. e.g. HCT240 is hematocrit at 240 days.

2 Chromosomal locations of top SNPs.

3 Positions of the top SNPs according to *Sus scrofa* Build 10.2 genome assembly.

4 The number of genome-wide significant SNPs for each hematological trait.

5 The associated interval was defined as the region in which the distance between any two neighboring genome-wide significant SNPs was less than 10 Mb.

6 Annotated genes nearest to the top SNPs.

### Loci for Erythrocyte Traits

**Figure 1:**
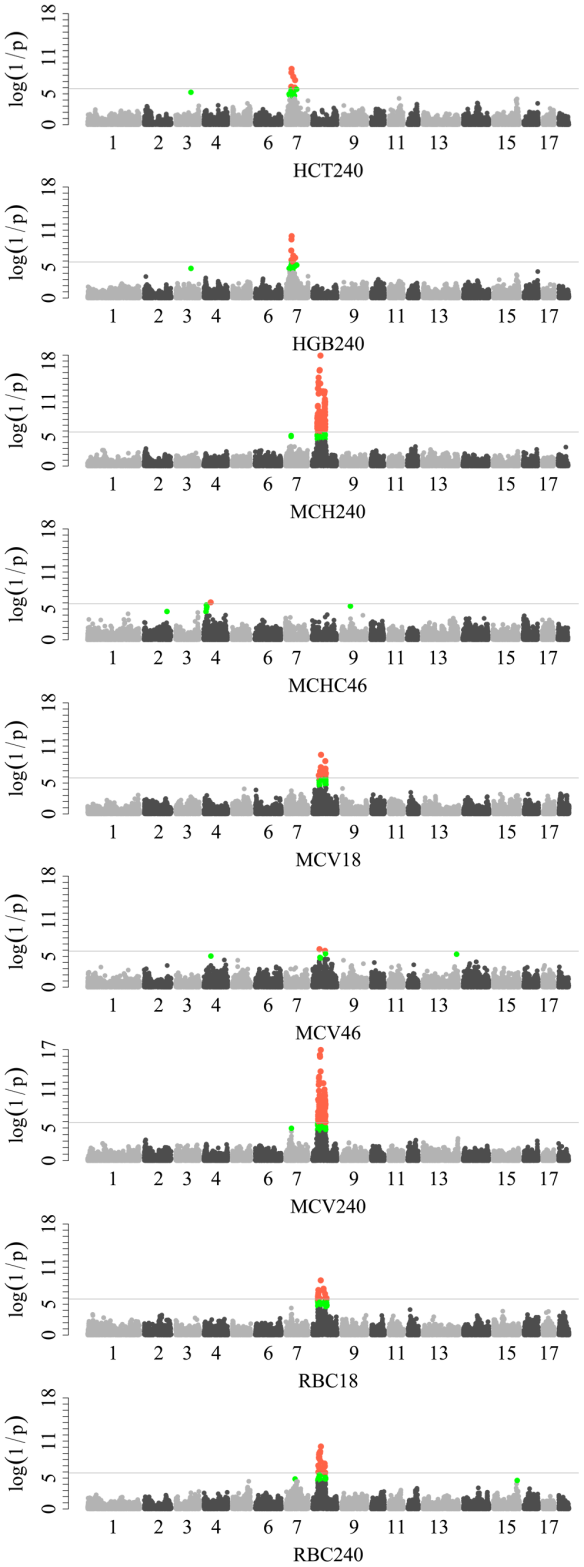
Manhattan plots for the single-marker analysis of erythrocyte traits log_10_(1/p) values are shown for all SNPs that passed quality control. The dotted line denotes the Bonferroni-corrected genome-wide significant threshold. SNPs surpassing the genome-wide threshold are highlighted in pink and SNPs reaching the suggestive threshold in green. HCT240: hematocrit at 240 days; HGB240: hemoglobin at 240 days; MCH240: mean corpuscular hemoglobin at 240 days; MCHC46: mean corpuscular hemoglobin content at 46 days; MCV18, MCV46, MCV240: mean corpuscular volume at 18, 46 and 240 days; RBC18 and RBC240: red blood cell count and at 240 days.

In total, we found 140 and 118 SNPs significantly associated with 7 erythrocyte traits by single-marker GWAS and LONG-GWAS, respectively. The corresponding Manhattan plots are shown in **Figure 1** and **Figure S1**.

#### Single-marker GWAS for erythrocyte traits

For HCT and HGB, we identified 3 genome-wide significant loci on SSC7. The loci cover the region from 33 to 56 Mb and had the effect exclusively on the measurements at day 240. The lead SNPs within the regions are identical for the two traits. No genome-wide significant SNP was found for these traits at days 18 and 46. For MCH, MCV and RBC, all significant SNPs were detected on SCC8 which appeared to harbor two independent associated regions (32.36–50.1 Mb and 66.03–84.49 Mb). The regions showed constant effect on MCV across the three age stages whereas exhibited time-specific influence on MCH and RBC as the association disappeared on early-stage samples at day 46. Notably, a cluster of SNPs (>100) on this chromosome exhibited signals of strong association with MCV and MCH at day 240 with the top SNP (ss131369293) at 50096834 bp. For MCHC, only ss131260759 at 4248936 bp on SSC4 achieved the genome-wide significant level for the trait measured at day 46.

#### LONG-GWAS for erythrocyte traits

A total of 118 SNPs within 10 genomic regions showed strong association with erythrocyte-related traits by LONG-GWAS. The results confirmed the findings of single-marker GWAS for HCT on SSC7, and for MCV, MCH and RBC on SSC8. It indicates that these loci consistently regulate red blood cells at different stages. Moreover, we uncovered two novel loci for HGB on SSC1 and SSC12 with the lead SNPs at 65994430 (ss120021119) and 29107229 (ss131459230) bp on the two chromosomes, respectively. One new locus was identified for HCT at 85640695 bp on SSC11. Only one significant locus for MCHC was found at 4168738 bp on SSC10, which differed from the result from the single-marker GWAS.

### Loci for White Blood Cell Counts

**Figure 2:**
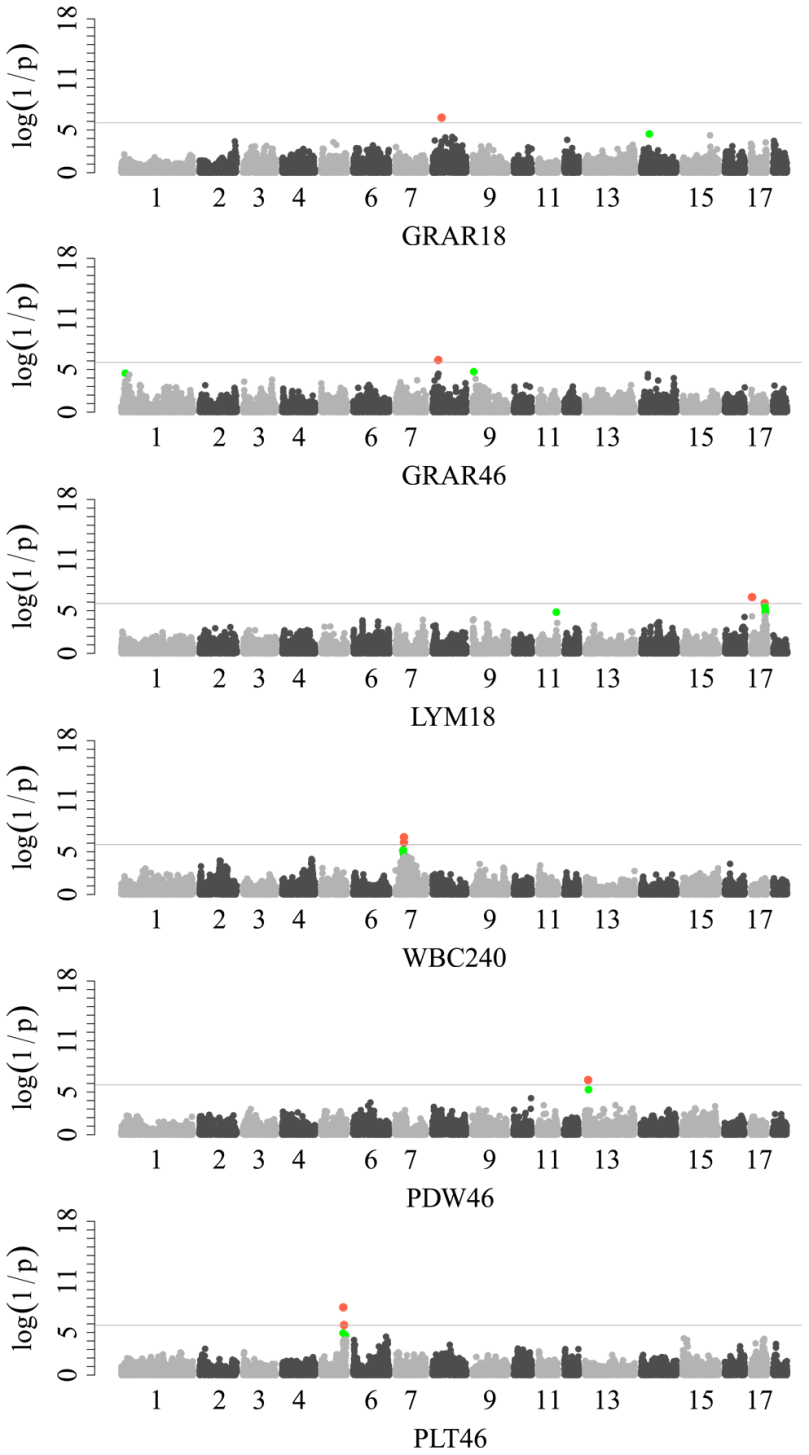
Manhattan plots for the single-marker analysis of white blood cell and platelet traits. log10(1/p) values are shown for all SNPs that passed quality control. The dotted line denotes the Bonferroni-corrected genome-wide significant threshold. SNPs surpassing the genome-wide threshold are highlighted in pink and SNPs reaching the suggestive threshold in green. GRAR18 and GRAR46: granulocyte count percentage at 18 and 46 days; LYM18: lymphocyte count at 18 days; WBC240: white blood cell count at 240 days; PDW46: platelet distribution width at 46 days; PLT46: plateletcrit at 46 days.

Only 6 genome-wide significant SNPs on 4 autosomes were identified for leukocyte traits by single-marker GWAS (**Figure 2**). Two SNPs within the *RAB44* gene on SSC7 were associated with WBC at day 240. Two significant SNPs (ss131368550 and ss131364780) for GRAR at days 18 and 46 were found at different positions (40.78 Mb and 26.31 Mb) on SSC8. Moreover, one SNP (ss131544979) at 2971217 bp on SSC17 was associated with LYM at the age of 18 days. A single SNP (ss478935524) at the position of 5860648 bp on SSC18 was associated with LYMA by LONG-GWAS.

### Loci for Platelet Traits

Analysis of platelet traits revealed two significant loci on SSC13 and SSC5 by single-marker GWAS and 39 associated loci by LONG-GWAS (**Figure 2**). In simple GWAS results, ss131296370 at 95971272 bp on SSC5 and ss107854351 at 14848132 bp on SSC13 were associated with PLT and PDW at day 46 with *P*-values of 1.14 E-8 and 3.94 E-7 respectively. For LONG-GWAS, two significant loci for PDW were detected on SSC8. The lead SNPs at the two loci were SS131368505 at 40852645 bp and ss131371056 at 75662581 bp with a distance of 34.81 Mb, implying at least 2 loci controlling PDW on chromosome 8.

## Discussion

### GWAS versus QTL Mapping

We have previously performed genome scans on the F_2_ population using 183 microsatellites. We detected 46 genome-wide significant QTL for baseline erythroid traits, 8 for leucocyte-related traits and 6 for platelet-related traits. These QTL are distributed on SSC1, 2, 7, 8, 10, 12, 13, 15 and X [17], [18]. In the present study, we did not test the association of SNPs on chromosome X as the currently available GWAS statistical models are hard to handle the random inactivation situation on the sexual chromosome. By using single-marker GWAS or LONG-GWAS on the 60K SNP data, we confirmed the previously identified QTL on SSC7 and SSC8 accounting for 46% of the total detected QTLs, and uncovered two additional genome-wide loci for HCT and HGB on SSC11 and SSC12, respectively. QTL mapping studies in F_2_ populations were generally conducted by comparing the phenotypic difference between F_2_ individuals inheriting different alleles from the founder breeds under the assumption that QTL alleles were alternatively fixed in each founder breed of the F_2_ intercross. The advantage of this mapping strategy is that we can anchor genomic regions affecting phenotypic traits using sparse markers. However, it could result in false negative signals If QTL alleles are segregating within founder lines. Moreover, the confidence intervals of most QTL were larger than 20 cM. In our QTL mapping study, the smallest intervals were 3 and 4 cM for MCV and MCH at day 18 on SSC8, respectively [17]. In comparison, GWAS test the average phenotypic difference grouped by alternative alleles of high density markers and without any assumption; thereby it could identify significant signals even if QTL is not fixed in founder breeds. Moreover, GWAS could narrow down the confidence interval of QTL to small genomic regions. In the current study, the confidence interval for MCV at day 18 on SSC8 were 0.80 Mb based on the 1.5 Lods drop rule [34]. Only a handful of genes exist in such small regions.

### QTL Replication with Other Studies

Until now, there were only two very recent GWAS papers describing significant genomic loci for hematological parameters in pigs [15], [16]. Luo *et al*. [15] detected 62 genome-wide significant and 3 chromosome-wide significant SNPs associated with erythrocyte traits by performing GWAS on a Large White × Chinese Min F_2_ intercross. All significant SNPs were found on SSC7 and SSC8 except one SNP associated with RBC on SSC1 and two SNPs for RDW on SSC12. Our results confirmed all of these findings. Wang et.al [16] identified 111 SNPs including 10 genome-wide significant SNPs and 101 chromosome-wide significant SNPs for 15 hematological traits in 2 Western breeds and one Chinese synthetic breed. The 111 SNPs are distributed on all autosomes except for SSC7, SSC8 and SSC18. However, none of these SNPs were replicated in our study. The reasons for the inconsistence could be different genetic background of experimental populations in the two studies, the complex genetic basis of hematological related cells, and different trait recording s. Wang *et al.* [16] measured hematological traits on pigs at day 35 after immunized with classical swine fever vaccine at day 21.

### Time Constant and Specific QTL

The single-marker GWAS revealed that the significant locus on SSC8 was consistently associated with MCV measured at days 18, 46, and 240, suggesting that a common variant regulates MCV at the whole life stage. The constant effect of this locus on MCV was further confirmed by LONG-GWAS that treated MCV data at the three ages together and obtained the same finding as the single-marker GWAS. In contrast, ss131544979 at 2971217 bp on SSC17 showed time specific effect on LYM at day 18. SNPs within two different regions on SSC8 were associated with GRAR at days 18 and 46, respectively, indicating that distinct genes are involved in development stages of granulocyte cells. Time specific loci were also evidenced for PLT and PDW on SSC5 and SSC13 as the association signals were observed only from the data at day 46. A high proportion of SNPs on SSC7 and SSC8 were identified for erythroid traits and leukocytes traits at day 240. Notably, the SNPs on SSC7 for HCT, HGB and WBC had a significant effect only at day 240 (**Table 2**) and therefore can be viewed as a late-acting QTL. It should be noted that all significant SNPs on SSC7 were not located in the SLA region (24.7 Mb - 29.8 Mb), which is response for immune system and is related to a range of diseases.

### LONG-GWAS Analysis

Currently, standard GWAS (e.g. GenABEL) only utilized one time point for each individual. If QTL constantly control the traits during the whole life process, it is reasonable to assume that jointly analysis of data at all-time points may be more powerful than the single-time-point approach. We thus used LONG-GWAS that utilized multiple phenotype measurements for each individual as proposed by Furlotte et al. [29]. We not only replicated the linkage mapping results for hematological trait, but also identified five new QTL affecting HGB, MCHC, HCT and LYMA on chromosomes 1, 12, 10, 11 and 18, respectively. One disadvantage of LONG-GWAS is that the significant signal may be overwhelmed by putting all time point’s data together if QTL effects vary during time stage. In this study, 5 time-specific expressed QTL for MCH, MCHC, PLT, PWD and LYM at early stage from 18 to 46 days were identified by the singer-marker GWAS, whereas these loci were not detected by LONG-GWAS.

### Haplotype Analysis for Single SNP Associated with Measured Phenotypes

In the standard GWAS, a prominent locus is usually featured by a lead SNP and a cluster of surrounding significant SNPs within a genomic region especially in the F_2_ pedigrees, in which high LD extents are expected. However, only one genome-wide significant SNP was associated with GRAR at days 18 and 46 (**Table S2**). None of suggestive SNP was detected in the neighboring region of the top SNP. To test if the signal was false positive result or real association, we conducted a haplotype based GWAS for this trait (**Figure S2**). We showed that dozens of genome-wide SNPs in a large interval were uncovered for GRAR at day 46, and the position of the top SNP was exactly the same to the lead SNP in the single-marker GWAS. For GRAR at day 18, the most significant SNPs were moved to another position (86.8 Mb), but the second top marker was identical to the top SNP identified in the single-marker GWAS. The findings support the reliability of the single significant marker for GRAR.

### Plausible Candidate Genes at the Significant Locus on SSC8

In the present study, the most interesting finding is the major locus for multiple hematological traits on SSC8. More than half of the detected regions (14 regions) were located on SSC8 that were associated with 9 hematological traits. Hundreds of significant SNPs in a large single region of more than 45 Mb on SSC8 were identified for MCH and MCV at day 240. In contrast, two segments in the ∼45 Mb region appeared to be independently associated with the two measurements at day 18 as no significant SNP was found in the inter-segment region (**Table 2**). To investigate whether one or two loci affect MCV and MCH on this chromosome, we re-analyzed MCH and MCV data conditional on the allelic effect of the lead SNPs (**Figure S3**). For MCH at day 18, after controlling for the effect of ss131369293, the second significant region disappeared. Also, no SNP showed association with MCH and MCV at day 240 after correcting for the effect of the lead SNP (ss131369293). Moreover, the complete LD (*D*′ = 1) was observed for the two top SNPs (ss131369009 at 44.93 Mb and ss107827400 at 66.03 Mb) for MCV at day 240 in the two intervals within the ∼45 Mb region on SSC8. These observations support one major locus for the tested hematological traits on this chromosome. However, we can not rule out the possibility that two neighboring genes contribute to the phenotypic traits as the conditional analysis can not distinguish the effects of two adjacent loci due to LD.

We noticed that SNPs associated with MCH, MCV, RBC and GRAR at the early stage mainly reside in the region of 36.31 to 50.10 Mb. The stem cell growth factor receptor (*KIT*) gene around 43.55 Mb on this chromosome stands out as a compelling candidate gene as it is essential for the development of hematopoietic stem cells and has been highly expressed in hematopoietic cells [35], [36]. Several mutations in *KIT* have significant influence in RBC in the mouse [37]. Johansson *et al.* [38] showed strong association of *KIT* variants with erythroid traits in piglets. Moreover, Fésüs *et al.* [39] reported the mild effect of *KIT* on hematological parameters in adult pigs. Our observation of the *KIT* region associated with hematological traits reinforces the assumption [40] that the *KIT* gene has a significant effect on peripheral blood cell measures in pigs.

## Conclusion

In conclusion, a total of 185 genome-wide significant SNPs corresponding to 91 genes were identified for 18 hematological traits at the three growth ages in the White Duroc × Erhualian F_2_ intercross. These loci confirmed the previously identified QTL and showed both time constant and specific effects on the measured traits. Of these findings, the most prominent one was the genomic region between 32.36 and 84.49 Mb on SSC8 that is associated with multiple erythroid traits. The *KIT* gene on this chromosome appears to be a promising candidate gene. The findings improve our understanding of the genetic architecture of hematological traits in pigs. Further investigations are warranted to characterize the responsible gene(s) and causal variant(s) especially for the major loci on SSC7 and SSC8.

## Supporting Information

Figure S1 **Manhattan plots for the LONG-GWAS analysis of hematological traits.** log_10_(1/p) values are shown for all SNPs that passed quality control. The dotted line denotes the Bonferroni-corrected genome-wide significant threshold. SNPs surpassing the genome-wide threshold are highlighted in pink and SNPs reaching the suggestive threshold in green. HCT: hematocrit; HGB: hemoglobin; MCH: mean corpuscular hemoglobin; MCHC: mean corpuscular hemoglobin content; MCV: mean corpuscular volume; RBC: red blood cell; LYMA: lymphocyte count percentage; PDW: platelet distribution width.
(TIF)

Figure S2 **Manhattan plots for the hidden haplotypes analysis of GRAR at 18 and 46 days on SSC8 where only one SNP was associated with each trait in the single-marker analysis.** SNPs surpassing the genome-wide threshold are highlighted in pink and SNPs reaching the suggestive threshold in green.
(TIF)

Figure S3 **Manhattans plots for conditional GWAS of MCV.** (A-C) Results are shown for MCV at 18 (A), 46 (B) and 240 (C) days. Grey and blue dots denote the results for SNPs before and after controlling for the top SNP (ss131369293) at 50.10 Mb on SSC8, respectively. Grey lines represent the genome-wide significant threshold.
(TIF)

Table S1 **Genome-wide significant SNPs associated with hematological traits by LONG-GWAS.**
(DOC)

Table S2 **Simple statistic results for GRAR at 18 and 46 days classified by the genotypes of the top SNPs.**
(DOC)
