# Supplementary figures and images for: Genome-Wide Association Study Reveals Constant and Specific Loci for Hematological Traits at Three Time Stages in a White Duroc × Erhualian F2 Resource Population
Source: PLoS One. 2013 May 17;8(5):e63665. doi: 10.1371/journal.pone.0063665 (PMC3656948; doi:10.1371/journal.pone.0063665)

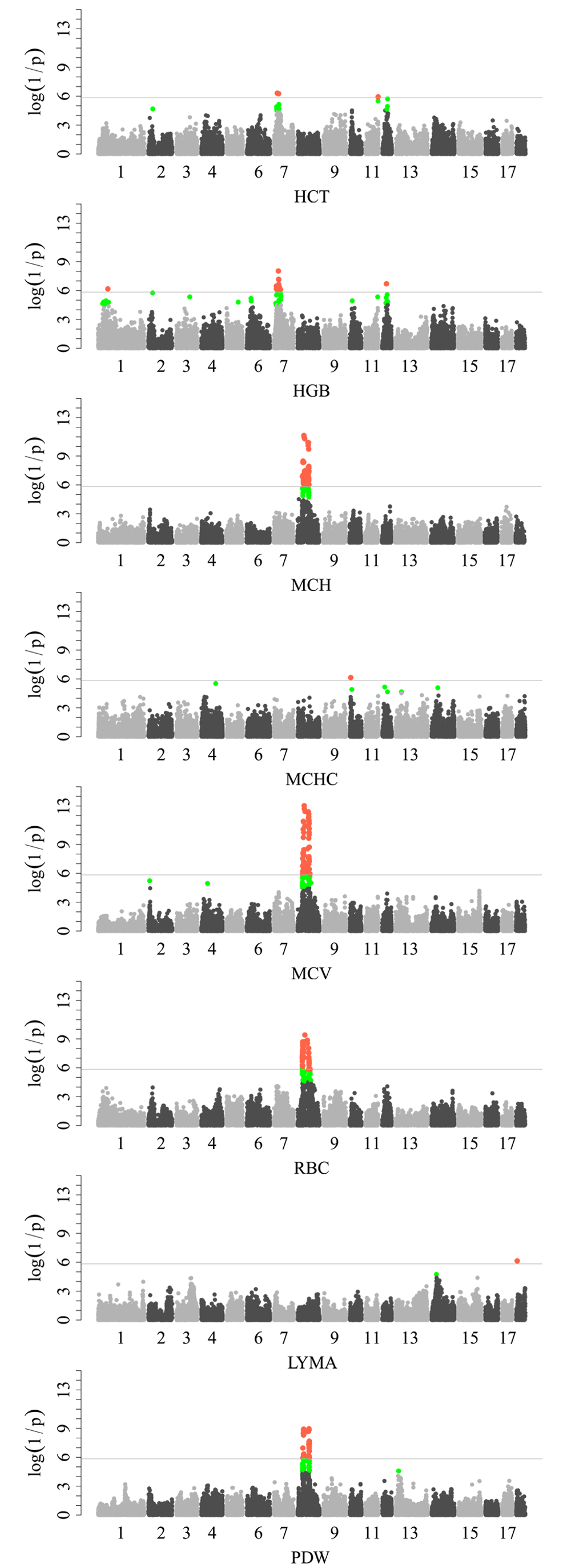

Supplement: Figure S1 — Manhattan plots for the LONG-GWAS analysis of hematological traits. log10(1/p) values are shown for all SNPs that passed quality control. The dotted line denotes the Bonferroni-corrected genome-wide significant threshold. SNPs surpassing the genome-wide threshold are highlighted in pink and SNPs reaching the suggestive threshold in green. HCT: hematocrit; HGB: hemoglobin; MCH: mean corpuscular hemoglobin; MCHC: mean corpuscular hemoglobin content; MCV: mean corpuscular volume; RBC: red blood cell; LYMA: lymphocyte count percentage; PDW: platelet distribution width. (TIF) [file pone.0063665.s001.tif]

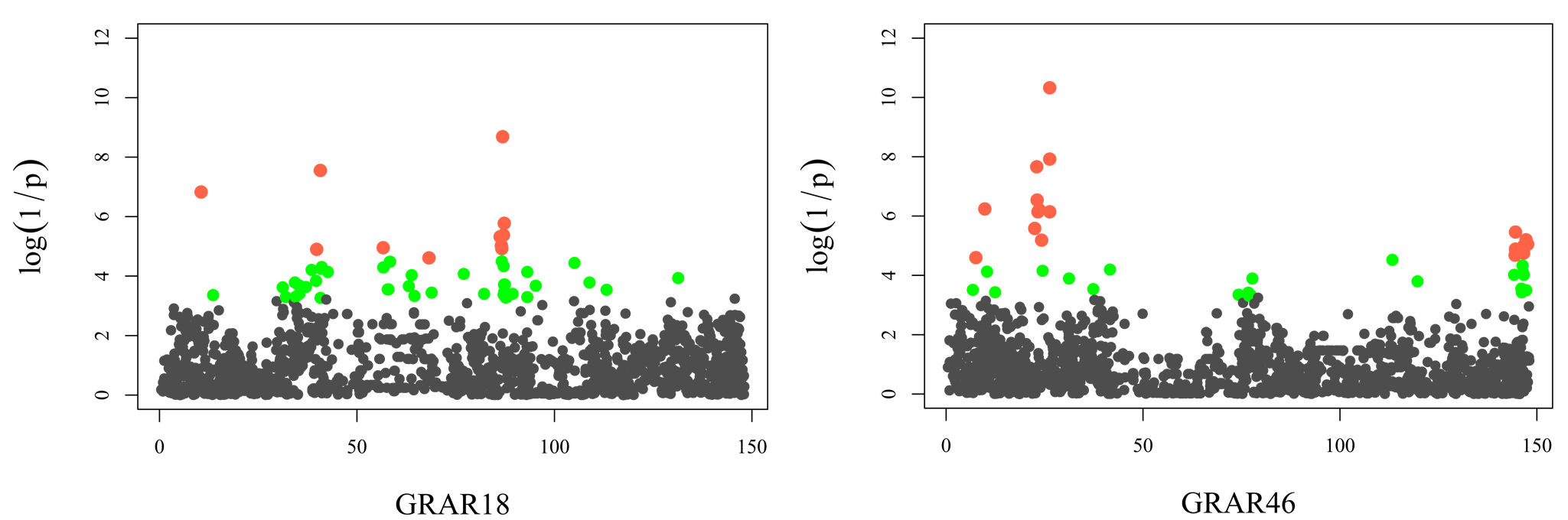

Supplement: Figure S2 — Manhattan plots for the hidden haplotypes analysis of GRAR at 18 and 46 days on SSC8 where only one SNP was associated with each trait in the single-marker analysis. SNPs surpassing the genome-wide threshold are highlighted in pink and SNPs reaching the suggestive threshold in green. (TIF) [file pone.0063665.s002.tif]

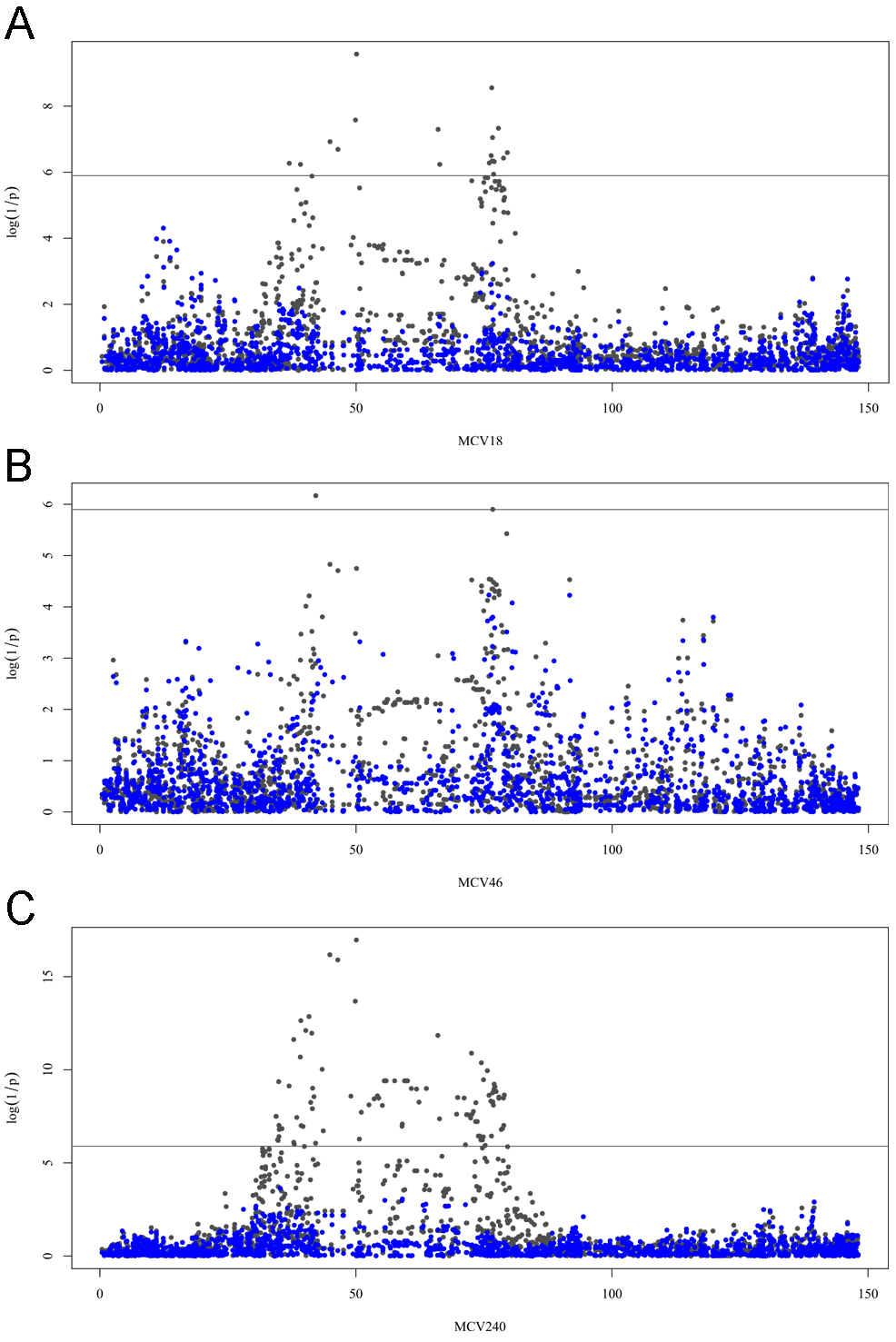

Supplement: Figure S3 — Manhattans plots for conditional GWAS of MCV. (A-C) Results are shown for MCV at 18 (A), 46 (B) and 240 (C) days. Grey and blue dots denote the results for SNPs before and after controlling for the top SNP (ss131369293) at 50.10 Mb on SSC8, respectively. Grey lines represent the genome-wide significant threshold. (TIF) [file pone.0063665.s003.tif]
