# Supplementary material for: Genome-Wide Association Study Reveals Constant and Specific Loci for Hematological Traits at Three Time Stages in a White Duroc × Erhualian F2 Resource Population
Source: PLoS One. 2013 May 17;8(5):e63665. doi: 10.1371/journal.pone.0063665 (PMC3656948; doi:10.1371/journal.pone.0063665)
Supplement: Table S1 — Genome-wide significant SNPs associated with hematological traits by LONG-GWAS. (DOC) [file pone.0063665.s004.doc]

Table S1. Genome-wide significant SNPs associated with hematological traits by LONG-GWAS

| Trait ^1^ | Top SNP | Chr ^2^ | Pos (bp) ^3^ | *P*-Value | Num_SNP ^4^ | Interval (Mb) ^5^ | Nearest Gene ^6^ |
| --- | --- | --- | --- | --- | --- | --- | --- |
| HCT | ss131338218 | 7 | 21815831 | 4.85E-07 | 2 | 21.82 - 31.03 | *SLC17A4* |
| HCT | ss131455151 | 11 | 85640695 | 1.17E-06 | 1 | 85.64 - 85.64 | *TUBGCP3* |
| HGB | ss120021119 | 1 | 65994430 | 6.71E-07 | 1 | 65.99 - 65.99 | *7SK* |
| HGB | ss131341609 | 7 | 31027719 | 9.32E-09 | 15 | 17.05 - 45.42 | *TINAG* |
| HGB | ss131459230 | 12 | 29107229 | 1.94E-07 | 2 | 29.02 - 29.11 | *CA10* |
| MCH | ss131369009 | 8 | 44927836 | 7.01E-12 | 95 | 34.9 - 79.19 | *TLL1* |
| MCHC | ss131567944 | 10 | 4168738 | 7.36E-07 | 1 | 4.17 - 4.17 | *FAM5C* |
| MCV | ss131369009 | 8 | 44927836 | 9.28E-14 | 95 | 34.39 - 84.49 | *TLL1* |
| RBC | ss131094241 | 8 | 49881116 | 4.00E-10 | 29 | 31.09 - 50.1 | *RXFP1* |
| RBC | ss478935224 | 8 | 66349700 | 1.37E-09 | 27 | 66.03 - 85.12 | *TECRL* |
| LYMA | ss478935524 | 18 | 5860648 | 7.13E-07 | 1 | 5.86 - 5.86 | *GALNTL5* |
| PDW | ss131368505 | 8 | 40852645 | 1.18E-09 | 13 | 34.39 - 50.1 | *OCIAD1* |
| PDW | ss131371056 | 8 | 75662581 | 1.07E-09 | 26 | 66.03 - 79 | *PPEF2* |

^1^ Abbreviations of hematological traits are given in Table 1. e.g. HCT240 is hematocrit at 240 days.

^2^ Chromosomal locations of top SNPs.

^3^ Positions of the top SNPs according to *Sus scrofa* Build 10.2 genome assembly.

^4^ The number of genome-wide significant SNPs for each hematological trait

^5^ The associated interval was defined as the region in which the distance between any two neighboring genome-wide significant SNPs was less than 10 Mb.

^6^ Annotated genes nearest to the top SNPs
