# Supplementary material for: Genome-Wide Association Study Reveals Constant and Specific Loci for Hematological Traits at Three Time Stages in a White Duroc × Erhualian F2 Resource Population
Source: PLoS One. 2013 May 17;8(5):e63665. doi: 10.1371/journal.pone.0063665 (PMC3656948; doi:10.1371/journal.pone.0063665)
Supplement: Table S2 — Simple statistic results for GRAR at 18 and 46 days classified by the genotypes of the top SNPs. (DOC) [file pone.0063665.s005.doc]

Table S2. Simple statistic results for GRAR at 18 and 46 days classified by the genotypes of the top SNPs

|  | Mean ± standard deviation (Number of individuals) | | |
| --- | --- | --- | --- |
| Genotype | 11 | 12 | 22 |
| GRAR18 | 3.67 ± 1.90 (13) | 8.32 ± 7.80 (280) | 10.92 ± 9.48 (478) |
| GRAR46 | 10.57 ± 2.12 (3) | 24.93 ± 15.07 (127) | 13.55 ± 12.56 (646) |
